# Artificial intelligence applications in CBCT-based assessment of craniofacial airway volume and shape in sleep-disordered breathing: a systematic review

**DOI:** 10.7717/peerj.21289

**Published:** 2026-06-17

**Authors:** Natheer H. Al-Rawi, Walid Elsayed, Sura F. Al-Bayati, Musab Hamed Saeed, Omar Abdul Jabbar Abdul Qader, Shishir Shetty, Asmaa Uthman

**Affiliations:** 1 Oral & Craniofacial Health Sciences, University of Sharjah, Sharjah, United Arab Emirates; 2 Department of Basic Medical & Dental Sciences, College of Dentistry, Gulf Medical University, Ajman, United Arab Emirates; 3 Department of Oral Biology, College of Dentistry, Suez Canal University, Ismailia, Egypt; 4 Diagnostic and Surgical Dental Sciences, College of Dentistry, Gulf Medical University, Ajman, United Arab Emirates; 5 Department of Clinical Sciences, College of Dentistry, Ajman University, Ajman, United Arab Emirates; 6 College of Dentistry, Al-Mashreq University, Baghdad, Iraq

**Keywords:** Artificial Intelligence, CBCT, SDB, OSA, Pharyngeal airway

## Abstract

**Background:**

Sleep-disordered breathing (SDB), including obstructive sleep apnea (OSA), is strongly influenced by craniofacial airway morphology. Cone-beam computed tomography (CBCT) enables three-dimensional airway assessment; however, manual segmentation is time-consuming and subject to variability. Artificial intelligence (AI) has emerged as a potential tool to automate airway analysis. This systematic review evaluates the technical performance and clinical applicability of AI in CBCT-based craniofacial airway assessment for SDB.

**Methods:**

This review was conducted in accordance with PRISMA 2020 guidelines and prospectively registered in PROSPERO (CRD 420251065028). A comprehensive search of PubMed, Ovid, Scopus, and Google Scholar was performed for studies published from 2015 onward. Eligibility criteria included diagnostic or validation studies assessing AI-based CBCT airway segmentation, landmark detection, or OSA prediction. Dual independent screening was conducted, and risk of bias was assessed using QUADAS-2.

**Results:**

Fourteen studies met the inclusion criteria. Most implemented deep learning architectures, particularly convolutional neural networks-based models such as U-Net and SpatialConfiguration-Net. Reported Dice Similarity Coefficients ranged from 0.90 to 0.97, and intraclass correlation coefficients for volumetric measurements typically exceeded 0.90, indicating strong agreement with manual segmentation. AI systems substantially reduced annotation time compared with manual methods. However, heterogeneity was considerable across AI architectures, anatomical targets, datasets, and outcome metrics. External validation was limited, and only one study evaluated OSA prediction against polysomnography.

**Conclusions:**

AI demonstrates high technical accuracy and efficiency in CBCT-based airway segmentation and morphometric analysis. Nevertheless, current evidence largely reflects internal technical validation rather than confirmed clinical utility. Additional prospective, multicenter studies incorporating clinical outcome validation are required before routine clinical implementation can be fully supported.

## Introduction

Sleep-Disordered Breathing (SDB) refers to a spectrum of respiratory abnormalities that occur during sleep, ranging from benign snoring to severe conditions like obstructive sleep apnea (OSA) (Memon & Manganaro, 2026). OSA is the most common type of SDB and is a major public health problem that affects almost a billion people around the world (Benjafield et al., 2019; Kandasamy & Almeleebia, 2023). It is marked by repeated episodes of partial or complete upper airway obstruction while sleeping, leading to intermittent hypoxia, fragmented sleep, and increased sympathetic activity. If left undiagnosed or untreated, OSA can lead to heart disease, high blood pressure, cognitive decline, metabolic dysfunction, and reduced quality of life (Benjafield et al., 2019; Schneider, 2023).

Craniofacial anatomy plays an important role in the development and severity of OSA. Changes in the shape of the maxilla, mandible, tongue, and soft palate can directly affect the dimensions and the ability of the upper airway to collapse (Kim & Kim, 2021; Neelapu et al., 2017). Therefore, imaging methods that can measure airway volume and shape are essential in both diagnosis and treatment planning. Cone-Beam Computed Tomography (CBCT) is a popular tool in dentistry and maxillofacial radiology due to its ability to provide high-resolution, three-dimensional visualization of craniofacial structures with relatively low radiation exposure (Al-Rawi et al., 2019; Aboudara et al., 2009) CBCT allows to measure the volume, cross-sectional area, and shape of the airway more accurately than traditional 2D lateral cephalograms (Aboudara et al., 2009)

Despite these advantages, manual interpretation, and segmentation of airway structures from CBCT scans takes a lot of time and effort, and subject to inter- and intra-observer variability (Uthman et al., 2021; Uthman et al., 2025). As a result, Artificial Intelligence (AI) has emerged as a promising adjunct in medical imaging. Using deep learning, especially convolutional neural networks (CNNs), AI algorithms can automatically detect, segment, and measure airway structures on CBCT images with high level of accuracy and efficiency (Orhan et al., 2022). This automation not only reduces human error but also accelerates diagnostic workflows and enhances reproducibility in clinical practice.

Additionally, advances in AI methodologies allow for detailed segmentation and analysis of airway morphology, helping to identify obstructive patterns that may not be easily detected through traditional imaging techniques (Sin et al., 2021; Naik, Mathew & Kundra, 2024). By using AI algorithms, clinicians can better assess airway characteristics, inform treatment decisions, and improve the outcomes for patients with sleep-related breathing disorders.

Recent research has shown that AI-assisted airway assessment is becoming more accurate and reliable (Ismail et al., 2024; Zhang et al., 2025; Wang, Xia & Jiang, 2021; Guo et al., 2025; De Rosa et al., 2025) For instance, Tsolakis et al. (2022) found a strong link between manual and AI-based measurements of airway volume and morphology, highlighting AI’s potential to serve as a useful diagnostic tool in sleep medicine (Tsolakis et al., 2022). Orhan et al. (2021) also tested an AI-based segmentation model that accurately identified pharyngeal airway narrowing on CBCT images. This suggests its applicability in detecting anatomical risk factors for OSA (Orhan et al., 2022; Ravelo et al., 2024).

This review is primarily intended for oral and maxillofacial radiologists, dental and sleep medicine clinicians, and researchers working in artificial intelligence–based medical imaging, with relevance for biomedical engineers and educators involved in AI-enabled airway assessment.

The goal of this review was to find out how accurately and efficiently AI can analyze CBCT images to evaluate airway volume and shape in patients with sleep-disordered breathing, especially obstructive sleep apnea. It compared AI performance to manual methods and explored its potential for improving diagnosis and treatment planning.

## Materials and Methods

This systematic review was conducted in accordance with the Preferred Reporting Items for Systematic Reviews and Meta-Analyses (PRISMA 2020) guidelines (Page et al., 2021). A review protocol was registered prospectively in the PROSPERO database (ID: CRD 420251065028) to ensure methodological transparency and avoid duplication. This addressed the following research question: “*How accurate and clinically useful are artificial-intelligence (AI) tools for CT/CBCT-based evaluation of craniofacial airway volume and shape in patients with sleep-disordered breathing*?”

The review question was structured according to the PICOS framework (Population, Intervention, Comparator, Outcomes and Study design) (Saaiq & Ashraf, 2017). The Population comprised patients who underwent craniofacial airway imaging using CT or CBCT imaging. The Intervention was AI-driven analysis for assessment or detection of sleep-disordered breathing. The Comparator consisted of conventional manual or clinician-based assessment methods The Outcomes- included diagnostic accuracy measures and other clinical performance metrics of AI based tools with traditional approach. Eligible study designs were diagnostic-accuracy or validation studies.

### Literature search strategy

A comprehensive literature search was conducted across four electronic databases: Ovid, Scopus, PubMed, and Google Scholar, in addition to Manual searching. To ensure the screening of all relevant articles, the search employed a set of keywords Boolean combinations of: (“artificial intelligence” OR “machine learning” OR “deep learning” AND “CBCT” OR “cone beam computed tomography” AND “airway” OR “upper airway” OR “pharyngeal airway” AND “sleep-disordered breathing” OR “obstructive sleep apnea” OR “OSA”).

The inclusion criteria limited studies to those published in the last 10 years (*i.e.,* from 2015 onward) to ensure that the review reflects the most current advancements in the field.

The search yielded a large collection of findings, methods, and perspectives, providing a full overview of the current research landscape in this area. The study included articles that were published in English and had full text available. It focused on AI-based assessment of diagnostic accuracy. The exclusion criteria ruled out non-English texts, abstracts, posters, and papers published before 2015.

### Study selection and data extraction

All records retrieved from the database searches were imported into Microsoft Excel for categorization and elimination of duplicates. The PRISMA 2020 framework directed the entire selection procedure. Following the elimination of 20 duplicates, 253 records were automatically designated as ineligible, and an additional 50 were eliminated for irrelevance to the review. Two independent reviewers (AU & NA) examined the remaining 508 records for eligibility using established criteria. Subsequent to this phase, 55 records advanced to full-text evaluation after the exclusion of 453 publications irrelevant to SDB.

**Figure 1:**
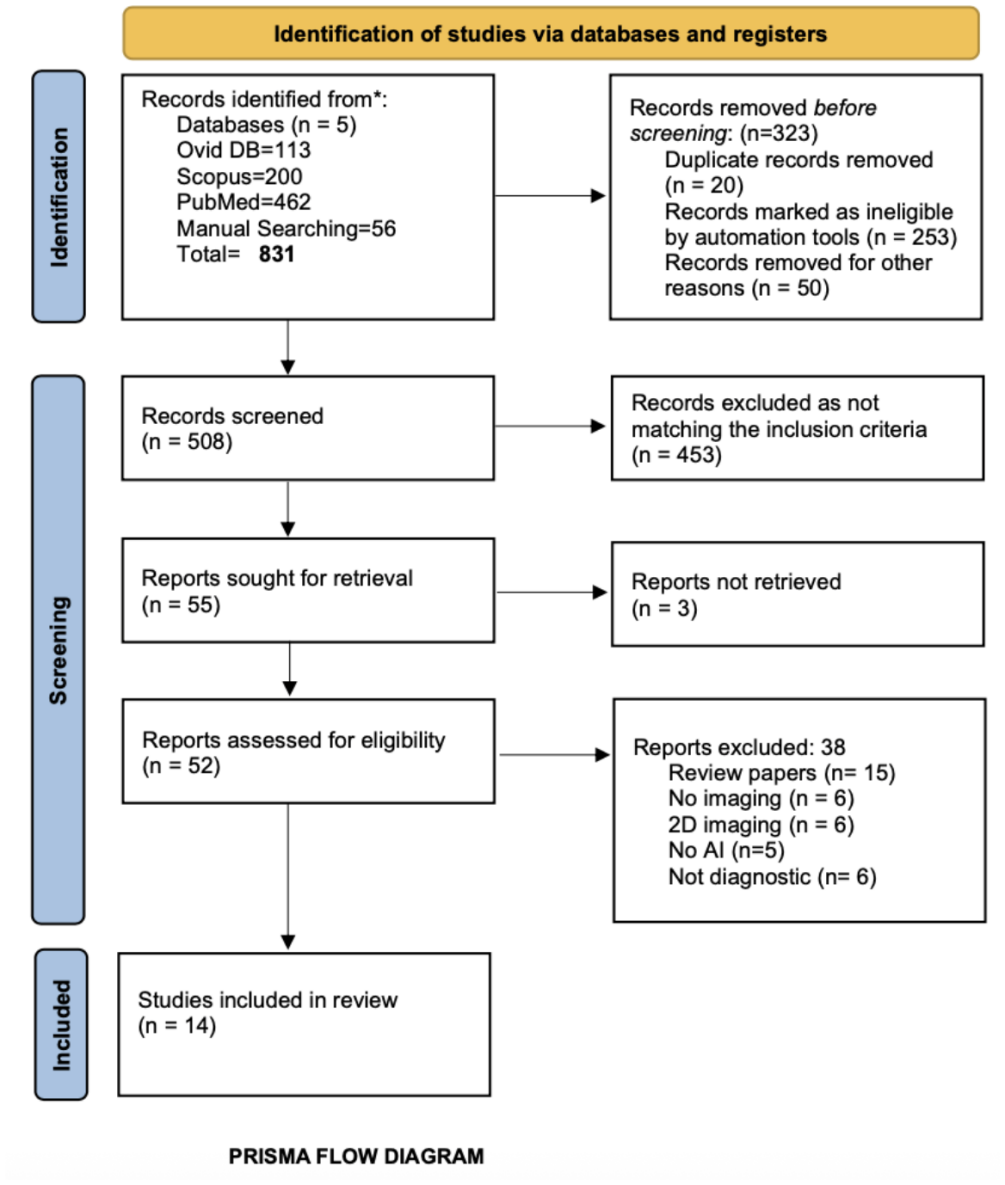
PRISMA flowchart.

Two reviewers individually assessed all full-text publications. Discrepancies between the two reviewers were reconciled through conversation with a third reviewer (SS). Fifteen studies were rejected for being review articles, six for employing 2D imaging, and seventeen for lacking imaging, artificial intelligence, or diagnostic applications. Three documents were inaccessible. In total, 14 papers fulfilled all inclusion criteria and were included in the qualitative synthesis (Fig. 1). Inter-rater reliability for title and abstract screening was assessed using Cohen’s κ, resulting in κ = 0.72, which signifies substantial agreement.

### Risk of bias

The Risk of Bias Assessment Tool (QUIDAS-2) was employed to evaluate the risk of bias and study quality for nonrandomized studies used to assess bias and applicability (Table 1). This tool looks at two main domains: the first pertains to the risk of bias, including patient selection, index test, reference standard, flow, and timing; the second addresses applicability concerns, which include patient selection, index test, and reference standard. This assessment help researchers in gauging the robustness and generalizability of the predictive models being analysed. The tool underscores the significance of external validation in model creation to reduce possible bias. Furthermore, the tool aids in pinpointing domains where bias may be present, allowing researchers to rectify these concerns and enhance the dependability of their prediction models. Carefully following the QUADAS-2 technique can enhance transparency and rigor in the assessment of prediction models, ultimately improving the quality of evidence utilized in clinical decision-making (Wade, Corbett & Eastwood, 2013).

**Table 1:** Risk of Bias.

	Risk of bias					Applicability concern		
	Authors (Year)	Patient selection	Index test	Reference standard	Flow & timing	Patient selection	Index test	Reference standard
1	Alsufyani et al. (2016)	Low	Low	Low	Low	Low	Low	Low
2	Zhang et al. (2019)	Low	Unclear	Low	Unclear	Low	Unclear	Low
3	Leonardi et at (2021)	Low	Low	Low	Low	Low	Low	Low
4	Liu et al. (2021)	Low	Low	Low	Low	Low	Low	Low
5	Shujaat et al. (2021)	Low	Low	Unclear	Low	Low	Low	Unclear
6	Sin et al. (2021)	Low	Low	Unclear	Low	Low	Low	Unclear
7	Cho et al. (2022)	Low	Low	Low	Low	Low	Low	Low
8	De Bataille et al. (2022)	Low	Low	Low	Low	Low	Low	Low
9	Dot et al. (2022)	Low	Low	Low	Low	Low	Low	Low
10	Orhan et al. (2022)	Low	Low	Low	Unclear	Low	Low	Low
11	Balashova et al. (2023)	Low	Low	Low	Low	Low	Low	Low
12	Chen et al. (2023)	High	Low	Unclear	High	High	Unclear	High
13	Chu et al. (2023)	Unclear	Low	Low	Low	Unclear	Low	Low
14	Kim et al. (2024)	Low	Low	Low	Low	Low	Low	Low

## Results

A total of 14 studies were included in this systematic review, all of which evaluated the performance of artificial intelligence (AI) algorithms for the automatic segmentation and analysis of the pharyngeal airway using cone-beam computed tomography (CBCT) in the diagnosis of SDB.

### Risk of bias and applicability concerns

The methodological quality of the included studies was evaluated using the QUADAS-2 tool. Out of the fourteen studies assessed, ten were considered to have an overall low risk of bias. These studies clearly described their patient selection criteria, applied the index test appropriately and independently of the reference standard, used valid reference standards, and ensured adequate flow and timing between assessments.

On the other hand, four studies showed concerns in one or more domains. One study was judged to have a high risk of bias due to insufficient methodological reporting in several areas. Three studies had unclear risk, primarily due to incomplete descriptions of patient recruitment methods, blinding procedures for the index test, or a lack of clarity regarding the reference standard used.

Most studies (12 out of 14) showed a low risk for patient selection by using well-defined inclusion criteria and representative samples. Two studies were giving a rating of unclear because they did not provide sufficient detail on the sampling method.

All but one study in the index test domain were rated as low risk. The majority provided adequate information to confirm that the index test was interpreted without knowledge of the reference standard. However, one study lacked clarity on whether blinding was maintained, resulting in an unclear rating.

Ten studies thought the reference standard was deemed appropriate, while the remaining four studies thought it was unclear. In these studies, reference method was either vague or not standardized without failed to describe them in sufficient detail.

Regarding flow and timing, many studies reported minimal delay between the index test and reference standard, and all or most participants received both assessments. One study was rated unclear due to insufficient reporting on the testing sequence and patient inclusion. Applicability concerns were low across nearly all studies. Thirteen studies were judged to have low concern in terms of how their patient populations, index tests, and reference standards aligned with the review question. One study was judged to have high applicability concern due to limited methodological transparency and reporting (Chen et al., 2023). Overall, the assessment supports the methodological soundness of the included studies, though a few showed limitation (Table 1).

### Workflow efficiency

AI-based methods significantly outperformed manual segmentation in speed. For example, Liu et al. (2021) demonstrated that their AI framework, SkullEngine, reduced the total time for segmentation and landmark detection from approximately 12 h to under 3 min. Similar time-saving benefits were reported by Alsufyani et al. (2016) and De Bataille et al. (2022) with automatic and semi-automatic methods offering both efficiency and accuracy.

Beyond segmentation, some studies evaluated diagnostic and morphometric applications. A multimodal model combining CBCT imaging and clinical data achieved an accuracy of 91% and an area under the receiver operating characteristic curve (AUC) of 0.916 for predicting moderate-to-severe OSA (Kim et al., 2024). while symbolic regression was used to identify anatomical predictors of airway narrowing (De Bataille et al., 2022). Segmentation accuracy was occasionally reduced in anatomically complex regions, such as the epiglottic area (Chen et al., 2023), but overall agreement with reference standards remained high. Additional investigations demonstrated feasibility in pediatric populations (Balashova et al., 2023) accurate landmark localization using deep learning frameworks (Dot et al., 2022), validated semi-automated nasal airway segmentation (Zhang et al., 2019) and assessment of skeletal pattern-related airway differences (Shujaat et al., 2021). Collectively, these findings support the technical feasibility and efficiency of AI-based CBCT airway analysis under research conditions.

To enhance interpretability and address methodological heterogeneity among the included studies, the characteristics table has been reorganized according to the primary AI task performed (Table 2). Specifically, studies were grouped into three categories: (1) segmentation and volumetric analysis studies, which focused on automated airway delineation and morphometric quantification; (2) landmark detection studies, which evaluated AI-based localization of craniofacial anatomical landmarks relevant to airway assessment; and (3) diagnostic prediction studies, which aimed to classify obstructive sleep apnea severity or risk using imaging-derived features. This task-based classification allows clearer differentiation between purely technical validation studies and those assessing diagnostic performance, facilitating more coherent synthesis of outcomes and improving transparency regarding the current evidence base. Key performance metrics including dice similarity coefficient (DSC), intraclass correlation coefficient (ICC), and AUC are highlighted to allow direct comparison across studies within each task category.

**Table 2:** Charectestics of included studies.

2A: Segmentation & Volumetric Studies.						
Author (Year)	Sample size	AI model/ tool	Reference standard	Key performance metrics	Main findings	Limitations
Alsufyani et al. (2016)	10 CBCT	Semi-automatic Segura tool	Manual segmentation	ICC (Volume): 0.96; ICC (Surface area): 0.97	Strong agreement with manual segmentation; time-efficient	No disease- specific outcome
Zhang et al. (2019)	10 CBCT	Airway Segmentor (AS) Mimics 19.0 & INVIVO 5	Phantom gold standard	ICC: 0.899–0.966; Random error: 3.9 to 4.5%	Volumetric estimates close to gold standard	Small pilot study
Leonardi et al. (2021)	40 CBCT	CNN-based segmentation	Manual segmentation	ICC: 0.921; DSC difference: 3.3–5.8%	Comparable performance to experienced reader	No clinical outcome validation
Liu et al. (2021)	170 CT/CBCT	Multi-stage CNN (SkullEngine)	Manual annotation	DSC > 0.90; Landmark error below clinical threshold	Reduced processing time from 12 hrs to <3 min	Not airway- specific focus
Shujaat et al. (2021)	103 CT/CBCT	3D U-Net	Manual segmentation	DSC: 0.97; IoU: 0.93; Precision: 0.97; Recall: 0.96	Strong agreement across airway regions	No diagnostic utility assessed
Sin et al. (2021)	306 CBCT	Deep-learning CNN	Manual (ITK-SNAP)	DSC: 0.919; IoU:0993 Accuracy: 0.961	High segmentation accuracy	OSA status not reported
Cho et al. (2022)	216 CBCT	3D U-Net	Expert manual segmentation	DSC: 0.928 Precision: 0.925; Recall: 0.921	Reduced precision in hypopharyngeal region	No OSA validation
De Bataille et al. (2022)	60 CBCT	Semi-automated 3D volumetric	Manual segmentation	ICC > 0.7	Strong correlation with manual measures	Restricted population
Orhan et al. (2022)	200 adults (PSG-confirmed OSA *vs* controls)	CNN-based segmentation (Diagnocat)	Manual radiologist segmentation	ICC: 0.954–0.972	Strong volumetric agreement	No diagnostic outcome tested
Balashova et al. (2023)	40 pediatric	Diagnocat AI segmentation	Visual exam + CBCT	Significant airway differences between groups	Effective 3D visualization	Not benchmarked to PSG
Chen et al. (2023)	30 CBCT	Semi-automatic segmentation (Amira)	Phantom gold standard	ICC: 0.948–0.997; Overestimation: 1.1–3.1%	Reliable nasal cavity volume measurement	Semi-automatic, limited automation

**2B: Landmark Detection Studies.**						
Author (year)	Sample size	AI model	Reference standard	Key performance metrics	Main findings	Limitations
Dot et al. (2022)	198 CT	Spatial Configuration-Net (SCN)	Manual landmarking	Mean Radial Error: 1.0 ± 1.3 mm; SDR > 90% (2 mm threshold)	Comparable accuracy to manual cephalometry	No external validation
Cho et al. (2022)	250 CBCT	SCN-based 3D U Net landmark detection	Manual landmarking	DSC:0,.928 Localization error: 0.34–0.70 mm; ICC: 0.98–0.99	Accurate upper airway landmark detection	One acquisition machine

**2C: Diagnostic Prediction Studies.**						
Author (year)	Sample size	AI Model	Reference standard	Key performance metrics	Main findings	Limitations
Kim et al. (2024)	502 training/ 55 test	Multimodal AI classifier (AirwayNet-MM-H)	**Polysomnography (PSG)**	AUC: 0.910; Accuracy: 91%; Sensitivity: 89.9% Specificity: 93.5% F Score: 93.2%	Outperformed unimodal models; effective OSA classification	Retrospective; single ethnicity; requires prospective validation

### Segmentation and volumetric analysis studies

Eleven studies primarily evaluated automated segmentation and volumetric quantification of upper airway structures using CBCT or CT imaging (Table 2A). Most implemented deep learning architectures, particularly CNN-based models such as 3D U-Net, multi-stage CNN frameworks, or proprietary AI systems.

Across segmentation studies, reported DSC ranged from 0.90 to 0.97, indicating excellent spatial overlap between AI-generated and manually annotated airway contours. ICC values, when reported, were similarly high (up to 0.89–0.97).

Volumetric agreement with manual segmentation was strong, with ICC frequently exceeding 0.95, and in several studies surpassing 0.95, reflecting near-equivalence to expert measurements (Orhan et al., 2022; Chen et al., 2023; De Bataille et al., 2022).

Time efficiency was consistently improved. For example, Liu et al. (2021) reported reduction in processing time from approximately 12 h for manual segmentation to under 3 min using a multi-stage CNN framework.

Although segmentation accuracy was robust across most anatomical regions, some studies reported reduced performance in complex areas such as the hypopharyngeal and epiglottic regions, likely due to soft-tissue contrast limitations in CBCT imaging (Chen et al., 2023).

Importantly, most segmentation studies assessed technical accuracy rather than diagnostic outcomes, and validation was predominantly internal.

### Landmark detection studies

Two studies focused primarily on AI-based craniofacial landmark localization relevant to airway morphometric assessment (Table 2B). These investigations employed SpatialConfiguration-Net (SCN) architectures for automated three-dimensional landmark detection (Cho et al., 2022; Dot et al., 2022)

Reported mean radial localization errors ranged from 0.34 mm to 1.0 mm, with success detection rates exceeding 90% within clinically acceptable thresholds (≤2–3 mm). Inter-rater reliability was high, with ICC values between 0.96 and 0.99.

These findings suggest that AI-assisted landmark detection can achieve reproducibility comparable to experienced clinicians. However, these studies did not directly evaluate clinical endpoints such as OSA severity or treatment planning outcomes. External validation was also limited.

### Diagnostic prediction studies

Only one study (Kim et al., 2024) primarily evaluated AI for diagnostic classification of obstructive sleep apnea severity using imaging-derived features combined with clinical metadata (Table 2C).

This multimodal deep learning classifier (AirwayNet-MM-H) was validated against polysomnography (PSG), the clinical gold standard for OSA diagnosis. The model achieved an AUC of 0.916 and an overall accuracy of 83.6%, outperforming unimodal imaging-only models (Kim et al., 2024).

While these findings are promising, the study was retrospective, single-center, and conducted within a relatively homogeneous population. Prospective multicenter validation remains necessary before broader clinical generalization.

In general, the findings demonstrate that AI can accurately and efficiently segment upper airway structures in CBCT scans, with performance equivalent to or better than expert clinicians. In addition to airway volume assessment, AI offers diagnostic value through anatomical risk identification and potential integration into screening and treatment planning for SDB.

## Discussion

This systematic review synthesized current evidence on AI applications for CBCT-based assessment of craniofacial airway structures in SDB. Across fourteen included studies, AI systems predominantly deep learning models based on convolutional neural networks (CNNs) demonstrated consistently high technical performance in automated airway segmentation, volumetric quantification, and, in some cases, diagnostic prediction of OSA severity.

### Technical performance and reproducibility

Segmentation-focused studies reported strong spatial agreement with manual expert annotations, with Dice Similarity Coefficients frequently exceeding 0.90 and intraclass correlation coefficients for volumetric measurements commonly above 0.98. These findings indicate excellent reproducibility and minimal volumetric discrepancy relative to reference standards. Additionally, AI systems substantially reduced annotation time compared with manual segmentation, in some reports decreasing processing time from several hours to minutes. Such efficiency gains may enhance workflow scalability in high-volume clinical setting (Liu et al., 2021).

Landmark detection models, including SCN architectures, achieved sub-millimetric localization errors and high success detection rates, suggesting that automated cephalometric and airway-related morphometric analyses are technically feasible. Multimodal classifiers integrating imaging features with clinical metadata demonstrated promising accuracy for OSA severity classification (however, these remain limited in number (Cho et al., 2022; Dot et al., 2022).

Collectively, these findings support the technical robustness of AI-driven CBCT airway analysis, particularly for segmentation and morphometric quantification tasks (Chen et al., 2023).

Despite strong performance metrics, substantial heterogeneity was observed across studies. Included investigations differed in:

• **Task type:** segmentation, landmark localization, or diagnostic classification
• **Model architecture:** 2D *vs* 3D CNNs, U-Net variants, SCN models, semi-automatic tools, and multimodal deep learning frameworks
• **Population characteristics:** pediatric *vs* adult cohorts, orthodontic samples without confirmed OSA, and PSG-confirmed OSA populations
• **Imaging systems and acquisition protocols:** different CBCT machines and voxel resolutions
• **Outcome metrics:** DSC, IoU, ICC, mean radial error, AUC, accuracy, sensitivity, and specificity

This multidimensional heterogeneity precluded quantitative meta-analysis and necessitated qualitative synthesis. Importantly, landmark detection studies were included because accurate craniofacial landmark localization contributes directly to airway morphometric evaluation and may inform airway risk assessment; however, these studies differ conceptually from segmentation-only investigations.

Future research would benefit from harmonized outcome metrics, standardized reporting, and clearer differentiation between segmentation performance and clinically validated diagnostic performance.

### Technical validity versus clinical utility

A critical finding of this review is the distinction between technical validation and demonstrated clinical impact. While segmentation accuracy and reproducibility were consistently high, most studies relied on internal validation datasets from single institutions. External validation across independent cohorts and imaging systems was uncommon (Kim et al., 2024).

Furthermore, only one included study evaluated AI-based classification against polysomnography the current clinical gold standard for OSA diagnosis. The majority of studies assessed anatomical or volumetric endpoints without correlating AI outputs to treatment decisions, disease progression, or patient-centered outcomes (Kim et al., 2024)

AI-assisted CBCT airway analysis offers several potential advantages, including reduction of observer-dependent variability, significant improvements in time efficiency, enhanced standardization of volumetric measurements, and facilitation of large-scale morphometric screening. By minimizing manual segmentation steps and operator subjectivity, AI-driven approaches may improve reproducibility and scalability in both clinical and research settings. Nevertheless, routine clinical adoption requires rigorous validation. Prospective multicenter studies are necessary to establish external validity and generalizability across diverse populations and imaging systems. AI-derived measurements should be benchmarked against clinically meaningful endpoints, such as PSG-confirmed OSA severity, to confirm diagnostic relevance (Kim et al., 2024). Furthermore, the impact of AI-generated outputs on therapeutic planning and decision-making must be systematically evaluated. Finally, successful implementation depends on seamless integration within existing radiologic workflows to ensure practicality, efficiency, and clinician acceptance.

### Reporting standards and methodological transparency

The reviewed studies varied in reporting completeness. As AI integration in medical imaging advances, adherence to emerging reporting frameworks such as CONSORT-AI, SPIRIT-AI, TRIPOD-AI, and STARD-AI will be essential to ensure reproducibility, transparency, and clinical interpretability. Future CBCT-AI research should explicitly report dataset partitioning strategies, external validation procedures, model calibration, and error analysis in accordance with these guidelines.

### Explainable AI and adoption barriers

Explainability remains a critical barrier to clinical implementation. Most included studies focused on performance metrics without describing interpretability tools such as saliency maps or gradient-based activation mapping. Clinician trust, regulatory approval, and medicolegal accountability depend on transparent decision pathways. The integration of explainable AI frameworks may therefore represent an essential step toward safe clinical translation.

### Limitations & recommendations for future studies

This review has some limitations. First, heterogeneity across AI tasks, architectures, and outcome metrics precluded meta-analysis. Second, included studies were predominantly retrospective and single-center in design. Third, restriction to English-language publications may introduce language bias. Finally, the rapidly evolving nature of AI research means that newer models may emerge beyond the review timeframe.

### Future directions

To advance the field, future investigations should prioritize multicenter prospective validation studies to enhance generalizability and external validity. AI-derived outputs must be rigorously evaluated against established clinical gold standards and correlated with meaningful patient outcomes to ensure clinical relevance. Furthermore, reporting practices should be standardized in accordance with emerging AI-specific guidelines to improve transparency and reproducibility. The integration of explainable AI mechanisms is essential to enhance interpretability and clinician trust. Finally, future research should assess cost-effectiveness and real-world workflow integration to determine the practical feasibility of AI implementation in routine clinical settings.

## Conclusion

Artificial intelligence applications for CBCT-based assessment of craniofacial airway structures in sleep-disordered breathing demonstrate consistent technical performance in automated segmentation and volumetric measurement. Deep learning models generally show strong agreement with manual expert annotations and reduced processing time. However, the current body of evidence primarily reflects internal technical validation rather than confirmed clinical effectiveness. External validation across diverse populations and imaging systems remains limited, and few studies have evaluated AI performance against clinical reference standards such as polysomnography.

Accordingly, While AI demonstrates strong technical performance in airway segmentation and measurement, current evidence supporting improved clinical decision-making or patient outcomes remains limited. Future prospective and multicenter validation studies are required.

## Supplemental Information

10.7717/peerj.21289/supp-1 Supplemental Information 1 PRISMA 2020 Checklist

10.7717/peerj.21289/supp-2 Supplemental Information 2 Searched Databases

## Additional Information and Declarations

### Data Availability

The following information was supplied regarding data availability: This is a Systematic review/Meta-analysis.
