# Supplementary material for: Artificial intelligence applications in CBCT-based assessment of craniofacial airway volume and shape in sleep-disordered breathing: a systematic review
Source: PeerJ. 2026 Jun 17;14:e21289. doi: 10.7717/peerj.21289 (PMC13282944; doi:10.7717/peerj.21289)
Supplement: Supplemental Information 1 [file peerj-14-21289-s001.docx]

**PRISMA 2020 Checklist Mapping**

| PRISMA Item | Requirement | Location in Revised Manuscript |
| --- | --- | --- |
| Title (1) | Identify report as systematic review | Title page |
| Abstract (2) | Structured abstract including objectives, eligibility criteria, information sources, risk of bias, results, conclusions | Revised Abstract |
| Introduction (3–4) | Rationale and objectives | Introduction section |
| Protocol & Registration (24a) | Provide registration information | Methods (PROSPERO CRD420251065028) |
| Eligibility Criteria (5) | Inclusion/exclusion criteria | Methods – Eligibility Criteria |
| Information Sources (6) | Databases searched with dates | Methods – Literature Search Strategy |
| Search Strategy (7) | Full search strings | Supplementary File – Search Strategy |
| Selection Process (8) | Screening method, reviewers, consensus | Methods – Study Selection |
| Data Collection Process (9) | Extraction methods | Methods – Data Extraction |
| Data Items (10a–b) | Outcomes and variables extracted | Table 2 + Methods |
| Risk of Bias (11) | Assessment method (QUADAS-2) | Methods – Risk of Bias |
| Effect Measures (12) | Specify metrics (DSC, ICC, AUC, etc.) | Results section |
| Synthesis Methods (13a–f) | Describe qualitative synthesis and heterogeneity | Results & Discussion – Heterogeneity subsection |
| Reporting Bias (14) | Consider risk of bias across studies | Discussion – Limitations |
| Certainty Assessment (15) | Overall certainty discussion | Discussion – Technical vs Clinical validity |
| Study Selection (16a–b) | PRISMA flow diagram | Figure 1 |
| Study Characteristics (17) | Study summary table | Table 2 |
| Risk of Bias Results (18) | QUADAS-2 findings | Table 1 + Results |
| Results of Individual Studies (19) | Key metrics reported | Results section |
| Results of Syntheses (20a–d) | Narrative synthesis provided | Results section |
| Reporting Biases (21) | Discuss limitations in evidence base | Discussion |
| Certainty of Evidence (22) | Interpretation of evidence strength | Discussion |
| Discussion (23a–d) | Summary, limitations, implications | Revised Discussion |
| Funding (25) | Funding disclosure | Declarations section |
| Competing Interests (26) | Conflict statement | Declarations section |
